# Debunking myths of protocol registration

**DOI:** 10.1186/2046-4053-1-4

**Published:** 2012-02-09

**Authors:** Stephanie M Chang, Jean Slutsky

**Affiliations:** 1 Agency for Healthcare Research and Quality, 540 Gaither Road, Rockville, MD 20850, USA

**Keywords:** protocol, publicly accessible, registration, systematic review, transparency

## Abstract

Developing and registering protocols may seem like an added burden to systematic review investigators. This paper discusses benefits of protocol registration and debunks common misperceptions on the barriers of protocol registration. Protocol registration is easy to do, reduces duplication of effort and benefits the review team by preventing later confusion.

## Background

The Agency for Healthcare Research and Quality (AHRQ) Evidence-based Practice Center (EPC) Program has long been committed to posting systematic reviews and protocols publicly [1]. Developing and posting a protocol benefits the systematic review community and their stakeholders, as well as the individual review team. The protocol defines the scope of the systematic review as well as the methods that will be used to conduct the review, thus providing not only transparency of the review but the ability for the systematic review community to track what reviews are taking place and their scope.

Increasingly, systematic reviews are the expected foundation for health technology assessments, clinical practice guidelines, coverage decisions and quality measures. The May 2010 Institute of Medicine (IOM) report on 'Clinical Practice Guidelines We Can Trust' [2] highlights the increasing recognition of the role of systematic reviews for making evidence-based decisions. With this increasing demand for systematic reviews in decision making, it becomes even more important to increase transparency, reduce redundancy and leverage resources in conducting reviews.

## Main text

Before embarking on a systematic review, researchers and funders scan the field to see if other reviews have been completed or are in progress and if there is a need for a new review [3,4]. Unfortunately, because many individual groups do not post their intentions of conducting a systematic review or the review protocol, the EPC Program has started a review only to have one on the same topic and scope be published shortly after.

In order to be useful, protocol registration needs to reach a tipping point where it becomes the norm. However, outside of those required by funders or journal editors, most reviews are not registered because of perceived burdens or barriers or a lack of a centralized registration process. We provide some arguments to the contrary below.

### Myth

There is no easy way to make my systematic review protocol available to the public. Not everyone has their own website like the AHRQ EPC program.

### Fact

In 2011, the National Institute of Health Research launched PROSPERO [5], an international prospective register of systematic reviews that is freely available to all [6].

### Myth

It doesn't benefit my team or me and, in fact, my ideas may get scooped.

### Fact

As described above, there is actually a great need and demand for systematic reviews by health care decision makers, guideline developers and other groups. Registration of the protocol may alert guideline groups that a related review is being conducted and provide opportunities for collaboration with partners for implementing the results of the review. Far from encouraging others to conduct a review on the same topic, protocol registration may be analogous to 'staking a claim' on a topic and be more likely to reduce duplication and competition on a topic by others and save scarce resources.

### Myth

It takes too much time to develop a protocol and it only helps other reviewers.

### Fact

Although protocols take time to develop, they are especially important when a review is conducted by more than one person to reduce confusion and ensure that all investigators are working from the same work plan. Without having understood the scope and methods up front, projects risk later wasted effort resulting from miscommunication, confusion or unintended bias. Posting of a protocol also enhances confidence in the resulting report. By determining methods *a priori *and reporting transparently, reviewers will find that end-users have greater trust that the report was not changed to suit the preference of the authors.

## Discussion

AHRQ continues to make all systematic review protocols conducted by the EPC program publicly available on their website [7] to improve the transparency, quality and conduct of reviews, as well as to reduce duplication. The IOM report on 'Standards for Systematic Reviews' [3] also recommends posting systematic review protocols for public comment. The EPC program supports the intent to engage actively with end-users of systematic reviews to further enhance applicability and collaborations, posting topic-specific protocol elements, such as the key questions that define the scope of the review, at an earlier stage for public comment [5]. General methods for conducting systematic reviews are guided by the EPC Methods Guide for Conducting Comparative Effectiveness Reviews [8], which is also posted for public comment before being finalized at http://www.effectivehealthcare.ahrq.gov/methodsguide.cfm.

## Conclusion

Developing and registering protocols may seem like an added burden to systematic review investigators, but it is easy to do, reduces duplication of effort and benefits the review team by preventing later confusion.

## Competing interests

The authors fund systematic reviews. Registration of systematic review protocols would greatly enhance efficiency.

## Authors' contributions

SC drafted the manuscript with contribution and input from JS. Both authors read and approved the final manuscript.
